# The Convergence of Biology and Material Science: Biomolecule-Driven Smart Drug Delivery Systems

**DOI:** 10.3390/biom15101383

**Published:** 2025-09-28

**Authors:** Yaqin Hou, Xiaolei Yu

**Affiliations:** 1 Department of Pharmacy, Affiliated Hospital of North Sichuan Medical College, Nanchong 637000, China; houyaqin@nsmc.edu.cn; 2 School of Pharmacy, North Sichuan Medical College, Nanchong 637000, China; 3 College of Life Sciences, Wuhan University, Wuhan 430072, China

**Keywords:** smart drug delivery, stimuli-responsive materials, biomolecules, DNA nanotechnology, personalized nanomedicine

## Abstract

Biomolecule-driven smart materials represent a paradigm shift in pharmacology, transitioning drug delivery from a passive process to an active, programmable, and highly specific intervention. These systems, constructed from or functionalized with biological macromolecules such as nucleic acids, peptides, proteins, and polysaccharides, are engineered to sense and respond to specific pathophysiological cues or external triggers. This review provides a comprehensive analysis of this rapidly evolving field. We first delineate the fundamental principles of stimuli-responsive actuation, categorizing systems based on their response to endogenous (pH, redox, enzymes, ROS) and exogenous (temperature, light, magnetic fields) triggers. We then conduct an in-depth survey of the primary biomolecular architectures, examining the unique design space offered by DNA nanotechnology, the functional versatility of peptides and proteins, and the biocompatibility of polysaccharides. Key therapeutic applications in oncology, inflammatory diseases, and gene therapy are discussed, highlighting how these intelligent systems are being designed to overcome critical biological barriers and enhance therapeutic efficacy. Finally, we address the formidable challenges—spanning biocompatibility, manufacturing scalability, and regulatory navigation—that constitute the “bench-to-bedside” chasm. We conclude by exploring future perspectives, including the development of multi-stimuli responsive, logic-gated systems and the transformative potential of artificial intelligence in designing the next generation of personalized nanomedicines.

## 1. Introduction

For decades, the paradigm of pharmacotherapy has relied on the systemic administration of therapeutic agents, a method fraught with inherent limitations. Conventional drug delivery systems (DDSs) frequently cause systemic adverse effects due to non-specific biodistribution of the active compound and the indiscriminate action of the drug on both diseased and healthy tissues [1]. This is particularly pronounced in cytotoxic cancer chemotherapy, where patients frequently suffer from severe cytotoxic side effects, limiting their treatment options [2,3]. Beyond this, many potent therapeutic molecules are hampered by poor physicochemical properties, such as low aqueous solubility or short in vivo half-lives, which compromise their ability to reach the target site at a therapeutically relevant concentration [4,5]. Consequently, maintaining a drug concentration within the therapeutic window often requires high and frequent dosing, which exacerbates systemic toxicity and undermines treatment efficacy [6].

To address these shortcomings, the field of drug delivery science emerged, initially focusing on passive carrier systems like liposomes and polymeric nanoparticles to improve drug solubility and stability [7]. A revolutionary conceptual leap, however, occurred with the development of “smart” or “stimuli-responsive” materials. These advanced materials are engineered to be dynamic and interactive, possessing the remarkable ability to undergo significant changes in their physicochemical properties in response to slight alterations in their surrounding environment [8]. This responsiveness enables “on-demand” drug release, where the delivery system remains stable until it reaches the specific microenvironment of a diseased tissue, which presents a unique biological stimulus. This capacity for spatiotemporal control—releasing the right drug, at the right place, at the right time—forms the cornerstone of modern precision medicine, promising to maximize local therapeutic efficacy while minimizing systemic exposure and toxicity [9].

While the first generation of smart materials was often based on purely synthetic polymers, the field is increasingly turning to nature’s own building blocks—biomolecules—as the core components for constructing the next generation of intelligent systems [10]. Biomaterials such as polysaccharides, proteins, peptides, and nucleic acids offer inherent advantages that are difficult to replicate synthetically, including excellent biocompatibility and biodegradability [10]. More profoundly, biomolecules provide an unparalleled design space for achieving true therapeutic intelligence. Their specific, genetically encoded sequences and complex structures can be precisely engineered to perform functions far beyond simple stimulus-response, allowing for the creation of systems with exquisite molecular recognition and the capacity to perform complex, logic-gated operations [11]. This evolution represents a conceptual shift from merely “smart” materials to truly “intelligent” systems that can process multiple inputs and execute pre-programmed actions, mimicking the sophistication of biological systems themselves [12]. The specificity of base pairing in DNA, for example, allows for the bottom-up construction of nanorobots with addressable components and conditional activation, a level of control unattainable with traditional synthetic polymers [13].

This review will provide a systematic and comprehensive exploration of the landscape of biomolecule-driven smart DDSs. The discussion will first delineate the fundamental principles of stimuli-responsive actuation, categorizing the various endogenous and exogenous triggers exploited to control drug release. It will then offer a detailed survey of the major classes of biomolecular architectures used to construct these systems, including platforms based on nucleic acids, peptides, proteins, and polysaccharides. Subsequently, the application of these intelligent systems in key therapeutic frontiers—with a focus on oncology, inflammatory diseases, and gene therapy—will be examined. Finally, this review will critically assess the formidable challenges that hinder clinical translation and will project the future directions of the field, including the rise of multi-stimuli responsive systems and the integration of artificial intelligence (AI) in material design. This review is based on a comprehensive literature search conducted in major scientific databases, including PubMed, Scopus, and Web of Science, using keywords such as ‘smart drug delivery’, ‘stimuli-responsive materials’, ‘biomolecules’, ‘DNA nanotechnology’, ‘peptide delivery’, ‘protein carriers’, and ‘personalized nanomedicine’.

## 2. Principles of Stimuli-Responsive Actuation

### 2.1. Exploiting Endogenous Cues: Responding to the Pathophysiological Milieu

**Figure 1:**
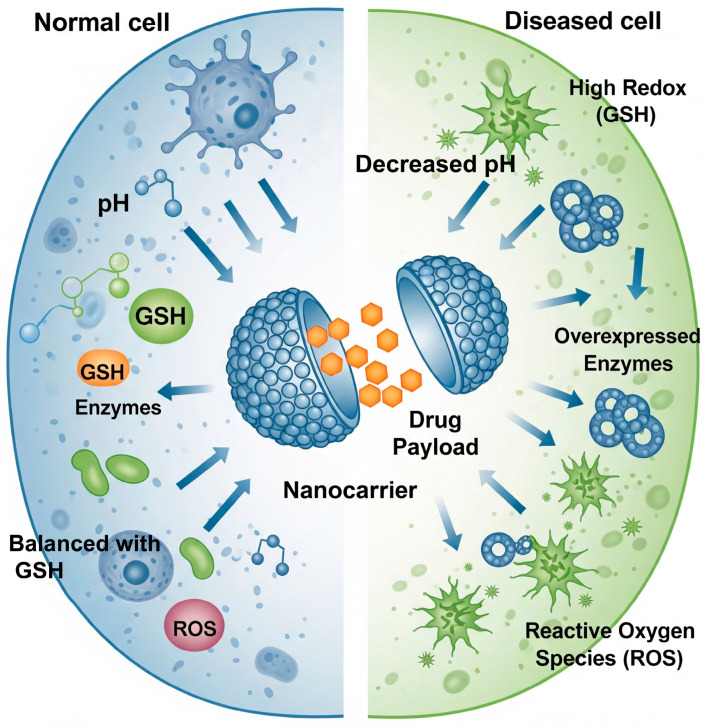
Smart nanocarriers responding to the pathophysiological milieu of diseased cells. The carrier remains stable under normal physiological conditions but is triggered to release its drug payload by the unique microenvironment of a diseased cell, characterized by decreased pH, high glutathione (GSH), overexpressed enzymes, and elevated reactive oxygen species (ROS).

The defining characteristic of a smart DDS is its ability to respond to a specific trigger. The choice of this trigger is a fundamental design decision that dictates the system’s mechanism of action, its potential applications, and its inherent advantages and limitations. These stimuli can be broadly categorized into two classes: endogenous cues, which are intrinsic to the body’s own physiological or pathological state, and exogenous triggers, which are applied externally to achieve precise spatiotemporal control. A thorough understanding of these actuation principles is essential for the rational design of effective therapeutic systems [14,15]. A fundamental dichotomy emerges in the design of these systems, representing a choice between biological autonomy and external control. Endogenous stimuli enable the creation of “autopilot” systems that can, in theory, navigate the body and activate automatically upon encountering the unique biochemical signature of a disease [6] (Figure 1). However, the precision of these systems can be compromised by the inherent biological heterogeneity of these cues, which can vary significantly between patients, disease types, and even within a single lesion [16]. Conversely, exogenous stimuli provide the clinician with a “remote control,” offering unparalleled precision over the location and timing of drug release. Yet, these methods are often constrained by physical limitations, such as the poor tissue penetration of light or the technical complexity of focusing energy fields deep within the body [8]. This central design tension between autonomy and control is a primary driver of innovation in the field, motivating the development of sophisticated multi-stimuli responsive systems that seek to combine the advantages of both approaches.

Smart systems designed to respond to endogenous stimuli are particularly appealing because they do not require external hardware for activation. They are engineered to recognize and react to the distinct biochemical and physiological hallmarks of diseased tissue, allowing for targeted drug release triggered by the pathology itself [16] (Figure 1). pH is one of the most commonly used triggers that exists between healthy and diseased tissues (Table 1). The extracellular pH in normal tissue and blood is usually maintained at around 7.4. However, because of high rate of glycolysis, the mean extracellular pH values in various solid tumors are usually below 7.0. Furthermore, pH difference can also be found in organelles such as in endosomes and lysosomes, in which the pH value is lower than other intracellular organelles with the pH range from 4.5 to 5.5 [6]. These gradients are reliable triggers for material designs such as ionizable polymeric systems, which change their charge, hydration, and conformation in response to pH shifts. A more precise mechanism involves acid-labile linkers, like hydrazones, which are stable at physiological pH but are hydrolytically cleaved in acidic conditions to liberate a conjugated drug [17,18]. The programmability of DNA has also enabled sophisticated pH-responsive devices, such as nanocontainers locked by an i-motif structure, a cytosine-rich sequence that folds and unlocks the device only in an acidic environment [19]. Due to the low pH of the tumor environment, histidine residues (pKa ≈ 6.0) are protonated, generating electrostatic repulsion; therefore, peptide-based nanostructures disassemble, facilitating endosomal escape via the proton sponge effect [20,21].

A second highly specific trigger is that the redox potential prevailing between extracellular (oxidative) and intracellular (reductive) space is associated with the intracellular glutathione (GSH) concentration of 2–10 mM (Table 1) [22]. This gradient is often even more pronounced in tumor cells and is primarily exploited through the incorporation of disulfide bonds (-S-S-), which are stable in circulation but are rapidly cleaved by intracellular GSH [23]. This cleavage can be leveraged in several ways: disulfide bonds can act as degradable cross-linkers to trigger the disassembly of a nanocarrier core; a drug can be covalently attached to a carrier via a disulfide linker for intracellular cleavage; or a shielding polymer layer like PEG can be tethered to a nanoparticle surface, allowing for de-shielding and activation of the nanoparticle after internalization [22]. The unique enzymatic fingerprint of diseased tissue provides another source of highly specific triggers, as conditions like cancer and inflammation are often characterized by the overexpression of enzymes like proteases and glycosidases [24]. A prominent example involves designing systems responsive to Matrix Metalloproteinases (MMPs) (Table 1), which are highly active in the tumor microenvironment (TME). By incorporating an MMP-cleavable peptide sequence, or the widely used cathepsin b-labile Gly-Phe-Leu-Gly (GFLG) motif, a nanocarrier can be designed to degrade and release its cargo, or even change its size to enhance tumor penetration, specifically in response to MMP activity [25]. Similarly, the enzyme hyaluronidase (HAase), which is also upregulated in the TME, can be used as a trigger. Nanoparticles coated with its substrate, hyaluronic acid (HA), benefit from a dual-function system where HA acts as both a targeting ligand for the CD44 receptor and as a gatekeeper that is enzymatically degraded upon internalization to release the drug [26]. This principle of enzyme-responsiveness extends to a wide range of other hydrolases, offering a broad toolkit for creating highly specific DDSs [27].

Finally, the localized oxidative stress found in inflamed tissues and tumors, characterized by an overproduction of reactive oxygen species (ROS) (Table 1), can be harnessed as an endogenous trigger [28]. ROS-responsive materials are designed by incorporating oxidation-labile moieties, such as thioethers or boronic esters, which are cleaved or change polarity in the presence of high ROS levels, leading to carrier destabilization [29,30,31]. A particularly elegant example is the use of bilirubin, a natural antioxidant, to form nanoparticles. When these nanoparticles accumulate at a site of inflammation, local ROS oxidizes and disassembles them, releasing an encapsulated drug while the bilirubin itself simultaneously scavenges ROS, providing a dual-mode therapeutic effect [32].

### 2.2. Harnessing Exogenous Triggers: External Control for Spatiotemporal Precision

External stimuli have the advantage of offering spatiotemporal control, since in these systems drug release is regulated by external factors, which can be precisely controlled in terms of location, duration, and intensity. Temperature is one of the most widely studied external triggers, typically exploited using polymers that exhibit a lower critical solution temperature (LCST). Below this temperature, the polymer is hydrophilic and soluble, while above it, the polymer undergoes a phase transition to become hydrophobic and aggregate [5]. The archetypal example is poly (N-isopropylacrylamide), which has an LCST of ~32 °C, conveniently close to physiological temperature. It is noteworthy that a variety of other polymers also exhibit LCST behavior, and their transition temperatures can be precisely tuned for biomedical applications through strategies like copolymerization [33,34]. A more advanced, bio-inspired alternative is elastin-like polypeptides (ELPs) (Table 1), whose transition temperature can be precisely controlled at the genetic level, offering superior monodispersity and biocompatibility [35]. The primary application for these materials is in conjunction with localized hyperthermia, where systemically administered, drug-loaded nanoparticles with a tuned LCST (~40–42 °C) are triggered to aggregate and release their cargo specifically within an externally heated tumor [16].

Light-responsive systems have the potential benefit of precision and can trigger drug release through either photochemical reactions or photothermal conversion (Table 1) [36]. Photochemical actuation involves incorporating photosensitive molecules like azobenzene, which undergoes a reversible, light-induced isomerization that alters its shape and polarity, thereby disrupting a carrier’s structure [37]. Alternatively, photocleavable linkers can be used to irreversibly release a conjugated drug upon light irradiation [16]. The photothermal approach utilizes nanomaterials, such as gold nanorods, that efficiently absorb near-infrared light—which has greater tissue penetration—and convert it into localized heat. This heat can be used for direct tumor ablation or to trigger release from a co-loaded thermo-responsive carrier [38,39]. However, considering the limitation of light wavelength for practical therapy, light penetration depth currently restrains the non-invasive applications for deep tissues.

To overcome the penetration limits of light, magnetic fields and ultrasound are used as non-invasive energy sources that can reach deep-seated tissues. Magnetic-responsive systems incorporate superparamagnetic iron oxide nanoparticles (SPIONs), which generate intense localized heat when subjected to an alternating magnetic field, a phenomenon known as magnetic hyperthermia (Table 1). This heat can then trigger release from a thermo-sensitive carrier, combining the deep penetration of magnetic fields with the sharp response of thermal triggers [40]. Ultrasound (Table 1), particularly high-intensity focused ultrasound, can trigger the release of drugs from carriers such as liposomes and micelles by localized heating or by mechanical effects like acoustic cavitation [16]. Furthermore, ultrasound can transiently increase the permeability of cell membranes and blood vessels (sonoporation), enhancing the local uptake of the therapeutic agent [41].

**Table 1:** Comparison of stimuli for responsive drug delivery.

Stimulus Type	Key Responsive Biomaterials/Moieties	Advantages	Limitations
pH [6,17,19]	Chitosan, poly (acrylic acid), histidine-rich peptides, DNA i-motifs, hydrazone/imine linkers.	Broad applicability (tumors, endosomes); autonomous activation.	Limited pH gradient between tumor/normal tissue; potential off-target activation in other acidic sites (inflammation).
Redox [16,22]	Disulfide cross-linkers, disulfide-conjugated drugs/polymers.	High specificity due to large intracellular/extracellular GSH gradient.	Primarily an intracellular trigger; less effective for extracellular release.
Enzymes [16,25]	Peptide sequences (MMP substrates), polysaccharides (hyaluronic acid), ester bonds.	Very high specificity and biological relevance; catalytic amplification.	Enzyme levels can be heterogeneous; potential for immunogenicity of peptide substrates.
ROS [28]	Bilirubin nanoparticles, polymers with thioether/thioketal linkers.	Targets oxidative stress characteristic of inflammation and cancer; can have dual therapeutic effect (drug delivery + ROS scavenging).	ROS levels can be transient and heterogeneous.
Temperature [35]	Elastin-like polypeptides (ELPs), poly(N-isopropylacrylamide) (PNIPAM).	Sharp, tunable response; can be triggered non-invasively with localized hyperthermia.	Requires external heating equipment; potential for damage to healthy tissue if heating is not precise.
Light [36,37]	Azobenzene, o-nitrobenzyl groups, gold nanoparticles, carbon nanotubes.	Unparalleled spatiotemporal control (on/off switching); non-invasive.	Limited tissue penetration depth, especially for UV/visible light; potential phototoxicity.
Magnetic Field [40]	Iron oxide nanoparticles (SPIONs) embedded in carriers.	Deep tissue penetration; enables dual imaging (MRI) and therapy.	Requires specialized equipment; potential for non-specific heating; guidance is limited to accessible sites.
Ultrasound [41]	Liposomes, micelles, nanoemulsions.	Non-invasive; deep tissue penetration; can enhance uptake via sonoporation.	Complex dose–response; potential for off-target tissue damage if not focused correctly.

## 3. Biomolecular Architectures for Intelligent Drug Delivery

### 3.1. Nucleic Acid-Based Nanotechnology: Programmability at the Molecular Level

**Figure 2:**
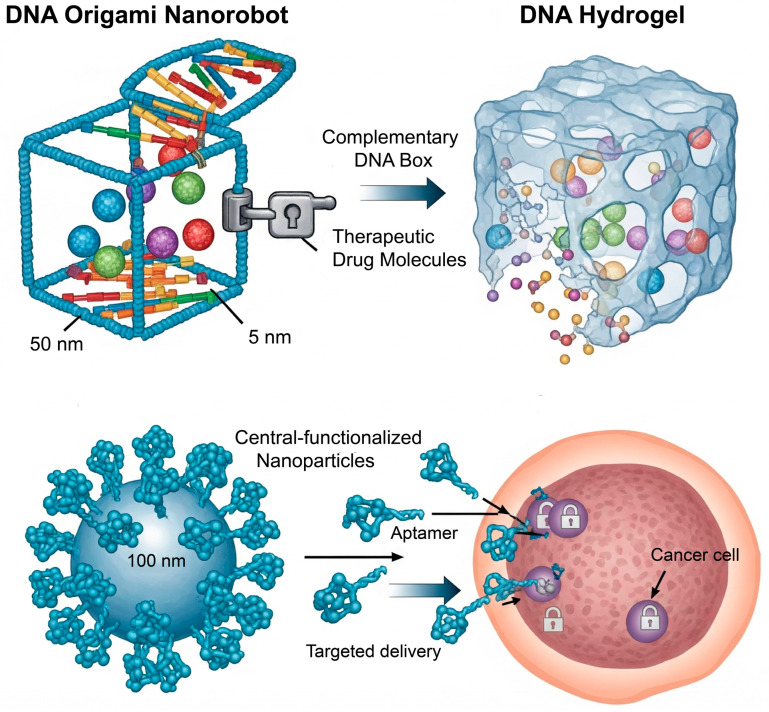
Versatile applications of DNA nanotechnology in drug delivery. (**Top left**) A DNA origami nanorobot for encapsulating and triggering drug release. (**Top right**) A DNA hydrogel for sustained release. (**Bottom**) Aptamer-functionalized nanoparticles for targeted delivery.

Among all biomolecules, nucleic acids—and DNA in particular—offer the highest degree of structural and functional programmability. The absolute specificity of Watson–Crick base pairing (A=T, G≡C) provides a simple yet powerful set of rules for designing self-assembling systems with nanoscale precision. This has given rise to the field of DNA nanotechnology, which treats DNA not just as a carrier of genetic information, but as a primary tool to create artificial structures suitable for numerous diverse applications (Figure 2) [42].

The unparalleled programmability of DNA, governed by the specificity of Watson–Crick base pairing, has established it as a premier material for bottom-up nanotechnology. This has led to the creation of precisely defined nanostructures through techniques like DNA origami, where a scaffold is folded into a pre-designed, predictable, addressable structure with various morphologies through several short staples [12]. The most striking feature of these nanostructures is their addressable surfaces, which allow precise spatial arrangement of therapeutic cargo and targeting ligands; thus, functional molecules can be accurately positioned at specific sites on the nanostructure [43]. The true power of this technology, however, lies in its capacity for dynamic actuation. By incorporating stimuli-responsive motifs, these static structures can be transformed into intelligent nanomachines. For example, a DNA box designed to carry a cytotoxic drug can be equipped with a lid held shut by a DNA duplex “lock” containing a pH-sensitive i-motif. Upon internalization into an acidic endosome, the i-motif folds, disrupting the lock and releasing the payload [44]. This concept has been extended to create sophisticated DNA nanorobots that function as logic-gated sensors, capable of remaining inert until they recognize a specific combination of cell-surface receptors, thereby triggering a conformational change that exposes a therapeutic agent only at the target cell (Figure 2) [45].

Beyond discrete nanostructures, DNA can also be cross-linked to form macroscopic, three-dimensional hydrogels for sustained therapeutic release [46]. The key feature of these hydrogels is that their structural integrity is programmable. The network can be designed to transition from a gel to a sol state in response to specific molecular triggers, releasing its entrapped cargo. This de-gelation can be initiated by introducing a “displacer” strand that competitively disrupts the network junctions, or by building stimuli-responsive elements directly into the cross-links. For instance, a hydrogel cross-linked by pH-sensitive i-motif structures will disassemble in neutral conditions, while one cross-linked by temperature-sensitive duplexes can be designed to “melt” at a specific temperature, demonstrating the versatility of this platform (Figure 2) [47].

In a functional rather than structural application, nucleic acids can be engineered to act as high-affinity binding agents known as aptamers. These short, single-stranded DNA or RNA oligonucleotides are selected in vitro through a process called SELEX to fold into unique conformations that bind to specific molecular targets with an affinity and specificity rivaling that of monoclonal antibodies [48]. In drug delivery, aptamers are primarily used as targeting ligands. By conjugating an aptamer to the surface of a nanocarrier, the system can be actively guided to cells that overexpress the aptamer’s target receptor [49]. Aptamers offer significant advantages over traditional antibodies, including lower immunogenicity, smaller size for better tissue penetration, and more reproducible and cost-effective chemical synthesis [48]. For example, the A10 aptamer, which targets the prostate-specific membrane antigen (PSMA), has been widely used to functionalize nanoparticles for delivering chemotherapy to prostate cancer cells, showing significantly enhanced efficacy in preclinical models (Figure 2) [48].

### 3.2. Peptide-Based Systems: Versatility in Structure and Function

Peptides, short chains of amino acids, occupy a unique and versatile space in the design of smart biomaterials. They bridge the gap between the digital, sequence-based programmability of nucleic acids and the bulk material properties of larger proteins and synthetic polymers. The specific sequence of a peptide can be engineered to dictate not only molecular recognition events but also the process of self-assembly into complex, functional nanostructures [50].

Peptides offer remarkable versatility as building blocks for smart materials, owing to their precise chemical structure, biocompatibility, and the ability to encode complex functions directly into their amino acid sequence. A major class of these materials is self-assembling peptides (SAPs), which are typically short, amphiphilic sequences designed to spontaneously organize in aqueous environments into well-ordered, hierarchical nanostructures such as nanofibers, micelles, or macroscopic hydrogels [51]. This assembly process, driven by a balance of non-covalent forces, creates hydrophobic cores that are ideal for encapsulating poorly soluble drugs, serving as biodegradable depots for sustained release [29]. Alongside SAPs, peptide–polymer conjugates represent another major class of materials, where peptides are covalently linked to synthetic polymers (e.g., PEG) to combine the biological functionality of the peptide with the advantageous physicochemical properties of the polymer, such as enhanced stability and circulation half-life [52,53].

**Figure 3:**
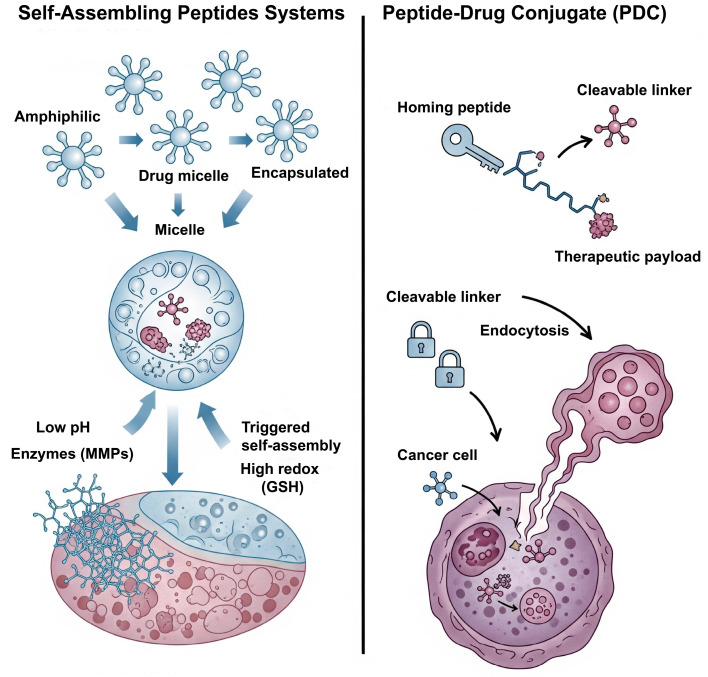
Dual strategies for peptide-based smart drug delivery. (**Left**) Self-assembling peptides form nanocarriers that release drugs in response to triggers in the tumor microenvironment. (**Right**) PDCs use a homing peptide for targeted delivery and intracellular release of a therapeutic payload.

The true “smart” nature of these systems emerges when stimuli-responsive elements are incorporated directly into the peptide sequence, making the self-assembly process itself a controllable event [54]. This inherent programmability allows for the design of nanostructures that disassemble in response to specific pathophysiological cues. For instance, pH-responsiveness can be conferred by including histidine residues, whose protonation in the acidic TME introduces electrostatic repulsion that drives disassembly [21]. Similarly, enzyme-responsiveness is achieved by engineering MMP-cleavable sequences into the peptide backbone, leading to carrier degradation specifically at sites of high enzymatic activity [29]. Furthermore, redox-responsiveness can be introduced via cysteine residues, which form stabilizing disulfide cross-links that are readily cleaved within the high-glutathione (GSH) environment of the cell, triggering intracellular drug release (Figure 3) [55].

Beyond their role in self-assembly, peptides are widely employed as targeting ligands to guide nanocarriers to specific cell types [56,57]. In this strategy, a short peptide with high affinity for a particular cell surface receptor is conjugated to the surface of a drug-loaded nanoparticle (e.g., a liposome or polymer micelle). A classic example is the Arginine-Glycine-Aspartic acid (RGD) peptide, which targets αvβ3 integrins that are overexpressed on angiogenic endothelial cells and many types of tumors [58]. By decorating a nanocarrier with RGD peptides, its delivery to the tumor site can be significantly enhanced. This approach separates the targeting function (the surface ligand) from the drug-carrying function (the nanoparticle core), offering great modularity in design.

In a distinct therapeutic strategy, peptides can function as highly specific targeting agents in the form of peptide-drug conjugates (PDCs), a clinically advancing modality [59]. A PDC typically consists of a ‘homing’ peptide that binds to a receptor overexpressed on target cells, a potent cytotoxic payload, and a cleavable linker connecting them (Figure 3) [60]. The peptide acts as a molecular guide, delivering the payload specifically to diseased cells, where it is internalized and the active drug is released. Compared to their larger antibody-based counterparts, PDCs offer advantages such as better penetration into solid tumors and lower immunogenicity, though their smaller size can lead to a shorter plasma half-life [61]. The clinical success of this platform is exemplified by Lutathera^®^, an FDA-approved PDC that uses a somatostatin analogue peptide to deliver a radioactive payload directly to neuroendocrine tumor cells. This concept has been further extended to peptide-drug-polymer conjugates, where a polymer chain is incorporated to improve solubility and pharmacokinetic profiles [62,63]. These tripartite constructs can self-assemble into nanostructures and often include cleavable linkers for controlled release.

### 3.3. Protein-Based Platforms: Leveraging Nature’s Workhorses

Proteins, as nature’s workhorses, represent a rich platform for drug delivery, leveraging their complex three-dimensional architectures, inherent biological functions, and excellent biocompatibility [64]. Several abundant, natural proteins have been successfully employed as drug carriers. Materials such as human serum albumin, gelatin, and silk fibroin are valued for their biocompatibility and biodegradability, allowing them to be formulated into nanoparticles or hydrogels that can physically encapsulate or non-covalently bind to therapeutic agents [64]. Albumin, the most abundant protein in blood plasma, serves as a natural transport vehicle, and nanoparticles derived from it can exploit endogenous uptake pathways (e.g., via the gp60 receptor) that are often upregulated in tumor cells. This principle is clinically exemplified by Abraxane^®^, an albumin-nanoparticle formulation of paclitaxel that enhances the drug’s solubility and tumor accumulation while eliminating the need for toxic excipients [65]. Similarly, other natural proteins like gelatin and silk fibroin are widely formulated into hydrogels and implantable devices for sustained, localized drug release, with silk fibroin being particularly noted for its exceptional mechanical strength and tunable degradation rate [66].

In addition to serving as bulk carriers, specific proteins are exploited as high-affinity ligands for active targeting. This strategy leverages the overexpression of certain receptors on diseased cells. For instance, the transferrin receptor is often upregulated in cancer cells to meet their high iron demand; consequently, conjugating the protein transferrin to a nanocarrier surface allows the carrier to hijack this natural uptake pathway for targeted delivery [67]. The most prominent examples of protein-based targeting ligands are antibodies, whose exquisite specificity has made them a cornerstone of targeted therapy. The conjugation of monoclonal antibodies to nanocarriers enables highly precise delivery to cells expressing the target antigen [68,69]. This concept is most clinically advanced in the form of antibody-drug conjugates, which, much like PDCs, link a potent cytotoxic drug directly to a targeting antibody, representing a powerful class of biotherapeutics.

While these natural proteins serve as effective carriers, recombinant protein engineering has enabled a more advanced, “bottom-up” approach, allowing for the creation of artificial proteins with precisely tailored, stimuli-responsive properties. ELPs are the premier example of this engineered strategy [35]. Produced recombinantly, ELPs have precisely defined amino acid sequences and molecular weights, which imparts a sharp and tunable inverse temperature phase transition. This behavior is characterized by a LCST, at which the polypeptides reversibly transition from soluble unimers to aggregated coacervates upon heating above a specific transition temperature (T_t_) [70]. This exquisite thermal control enables a range of sophisticated delivery strategies. For instance, ELPs with a Tₜ below body temperature can be injected as a solution that aggregates in situ to form a localized, biodegradable depot for sustained drug release over weeks or months [71]. In their most “smart” application, circulating ELP-drug conjugates with a Tₜ of ~40 °C can be triggered to accumulate specifically within a locally heated tumor, concentrating the therapeutic payload precisely where it is needed in response to externally applied hyperthermia [72].

## 4. Therapeutic Frontiers and Applications

### 4.1. Oncology: Targeting the TME

Cancer therapy remains the most prominent and intensely researched application for smart DDSs, largely because the TME presents a unique constellation of pathophysiological features—such as abnormal vasculature, acidity, and elevated enzyme levels—that can be exploited as triggers for targeted drug release [73]. A primary challenge is overcoming the biological barriers to delivering a sufficient drug concentration to the tumor. The foundation of nanoparticle delivery involves enhanced permeability and retention effect due to poor vascular structure and lymphatic drainage of diseased tissue (tumor) [74]. Later on, active targeting was achieved by attaching ligands (e.g., aptamers, peptides) on the surface of nanocarriers, which bind to receptors overexpressed on cancer cells, which leads to cellular uptake of nanocarriers [75]. Even after accumulation, the dense tumor stroma can impede deep tissue penetration. To address this, advanced systems are designed to be stimuli-responsive; for instance, a larger nanoparticle can be engineered to degrade into smaller, more penetrative fragments in response to MMPs in the TME, thereby achieving more homogenous drug distribution [76].

A major cause of chemotherapy failure is the development of multidrug resistance (MDR), often mediated by the overexpression of efflux pumps like P-glycoprotein (P-gp) that expel drugs from cancer cells [77]. Smart DDSs offer powerful strategies to circumvent MDR. By utilizing endocytosis for cellular entry, nanocarriers can sequester their payload within vesicles, effectively bypassing the efflux pumps located on the plasma membrane. Furthermore, systems designed for intracellular burst release—triggered by endosomal pH or cytosolic GSH—can overwhelm the pump capacity by releasing a high local drug concentration suddenly [78]. A highly effective approach also involves using a single nanocarrier to co-deliver the chemotherapeutic agent alongside an MDR inhibitor, such as an siRNA designed to silence the P-gp gene, ensuring both agents act on the same cell simultaneously [78].

Finally, smart nanocarriers are ideal scaffolds for theranostics, a paradigm that integrates therapeutic and diagnostic capabilities into a single agent [79]. A theranostic nanoparticle can be co-loaded with a therapeutic drug and an imaging contrast agent (e.g., SPIONs for MRI or quantum dots for fluorescence). This allows for non-invasive, real-time visualization of the nanocarrier’s biodistribution, providing invaluable feedback to confirm tumor accumulation and personalize treatment regimens. More advanced designs are “activatable,” where the imaging signal is only turned on by a tumor-specific stimulus, significantly improving diagnostic accuracy and the signal-to-noise ratio [80].

### 4.2. Inflammatory and Autoimmune Diseases

The pathological microenvironments of chronically inflamed tissues often share key biochemical hallmarks with the TME, including localized acidity, elevated levels of degradative enzymes, and high concentrations of ROS. This convergence means that many of the smart DDS platforms developed for oncology can be readily adapted for treating inflammatory and autoimmune diseases [81].

In rheumatoid arthritis, for example, the inflamed synovial joint is a site of intense oxidative stress, providing a specific trigger for targeted therapy [28]. A compelling strategy is the use of celastrol-loaded bilirubin nanoparticles (CLT/BRNPs). Celastrol is a potent anti-inflammatory compound limited by poor solubility and systemic toxicity. When encapsulated within nanoparticles self-assembled from PEGylated bilirubin, the system can accumulate in the inflamed joint [82]. The high local ROS levels then oxidize the bilirubin carrier, causing the nanoparticle to disassemble and release its celastrol payload. This design provides not only targeted delivery but also a dual therapeutic benefit, as the bilirubin itself acts as an antioxidant. Preclinical studies have shown that this system significantly enhances the anti-arthritic effect of celastrol while markedly reducing its systemic toxicity [82].

Similarly, inflammatory bowel disease presents unique targeting cues in the inflamed colonic mucosa, such as eroded mucus layers and high levels of degradative enzymes [83]. To exploit these features, an inflammation-targeting hydrogel has been developed for localized rectal administration. The hydrogel, formed from negatively charged microfibers of ascorbyl palmitate, preferentially adheres to positively charged inflamed lesions via electrostatic interactions [84]. The hydrogel is loaded with an anti-inflammatory drug like dexamethasone and is designed to be disassembled by the abundant esterase enzymes in the inflamed tissue. This enzymatic cleavage provides sustained, localized release of the drug directly at the ulcerated surface, a strategy shown to maximize local drug concentration and dramatically decrease systemic side effects compared to conventional therapies [85].

### 4.3. Gene Therapy: The Next Generation of Non-Viral Vectors

Gene-based therapies, which utilize nucleic acids like plasmid DNA (pDNA), messenger RNA (mRNA), or small interfering RNA (siRNA) to modulate gene expression, hold enormous potential for treating a vast array of diseases. A primary obstacle to their clinical use has been the development of safe and effective delivery vectors. While viral vectors are highly efficient, they carry risks of immunogenicity and insertional mutagenesis, making smart biomaterials a powerful emerging class of non-viral alternatives [86].

**Figure 4:**
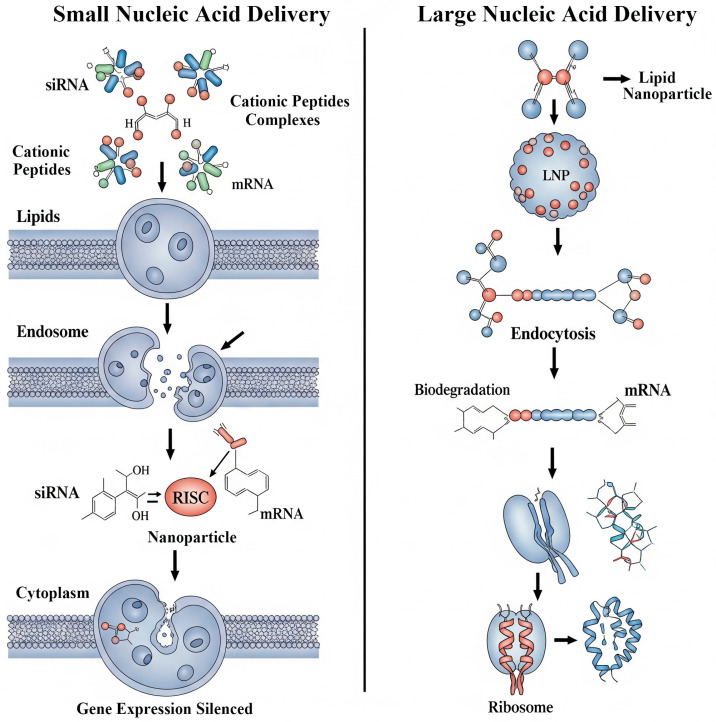
Non-viral delivery strategies for different nucleic acid therapies. (**Left**) Small nucleic acid (siRNA) delivery using cationic peptides to induce gene silencing. (**Right**) Large nucleic acid (mRNA) delivery using LNPs to enable protein production.

The delivery of small nucleic acids like siRNA and miRNA, which can trigger sequence-specific gene silencing, is challenged by their rapid degradation by nucleases and their inability to cross the anionic cell membrane (Figure 4) [87]. Smart peptide-based carriers are particularly well-suited to overcome these barriers. Cationic peptides, such as cell-penetrating peptides, electrostatically complex with the negatively charged siRNA, condensing it into a nanoparticle that facilitates cellular uptake. More advanced, multifunctional designs also incorporate endosome-disrupting domains to ensure the siRNA escapes into the cytoplasm and reaches its site of action in the RNA-induced silencing complex (Figure 4) [88].

Delivering larger nucleic acids like pDNA and mRNA presents even greater challenges, including the need for pDNA to cross the nuclear membrane (Figure 4). Cationic polymers (forming polyplexes) and lipids (forming lipoplexes) are the foundational platforms for their non-viral delivery, condensing the nucleic acid into a protected nanoparticle [86]. “Smart” versions of these vectors are engineered to be biodegradable, often incorporating disulfide or ester linkages that are cleaved intracellularly. This degradation is critical for reducing the cytotoxicity associated with persistent cationic charges and for facilitating the “unpacking” and release of the nucleic acid payload. The unprecedented success of the mRNA vaccines for COVID-19, delivered via sophisticated lipid nanoparticles (LNPs), has catalyzed a massive wave of research into advanced, smart vectors for a new generation of mRNA therapeutics and vaccines [89,90].

## 5. Translational Hurdles and Future Perspectives

### 5.1. The Bench-to-Bedside Chasm: Challenges in Clinical Translation

Although promising results have been achieved, in our view, there are still numerous scientific, technical, and regulatory obstacles ahead for smart DDS in their transition from proof-of-concept to clinical application [6]. A primary determinant of clinical viability is the material’s interaction with the complex biological system. The entire DDS and its degradation products must be rigorously evaluated for biocompatibility to ensure they do not elicit a harmful host response [91]. A major challenge is overcoming the body’s natural defense mechanisms, as nanoparticles are often rapidly cleared from circulation by the mononuclear phagocyte system [92]. While “stealth” coatings like PEG can mitigate this, they can sometimes trigger an immune response upon repeated administration [93]. Furthermore, upon entering the bloodstream, a nanoparticle is immediately coated with a dynamic layer of proteins, forming a “protein corona” that becomes its new biological identity. This corona can mask targeting ligands and mark the particle for immune clearance, representing a significant obstacle in nanomedicine [94].

Beyond biological barriers, the challenges of manufacturing and scalability are a major bottleneck [95]. Many synthesis methods developed in academic labs are difficult to perform reproducibly and even harder to scale up from milligram to kilogram quantities required for clinical use. Maintaining precise control over critical quality attributes like particle size and drug loading during scale-up is a non-trivial engineering challenge [96]. To address this, advanced manufacturing techniques like microfluidics are being developed, which allow for the continuous and highly controlled production of monodisperse nanoparticles with excellent batch-to-batch reproducibility [96]. Concurrently, the multi-component nature of smart DDSs makes their characterization and quality control exceptionally complex, requiring a sophisticated and expensive suite of analytical techniques [97].

Finally, the novel and complex nature of smart DDSs presents unique challenges for navigating the regulatory landscape. These systems are often classified as “combination products”—consisting of both a drug and a device component—which can complicate the review process at agencies like the FDA [98]. A significant hurdle is the lack of established, standardized guidelines for evaluating these diverse and sophisticated systems, creating uncertainty for developers [6]. While regulatory bodies are actively working to develop new frameworks for nanomedicines, navigating this evolving landscape remains a significant challenge for bringing these promising technologies to patients [93].

### 5.2. The Future of Smart Delivery: Towards Greater Complexity and Control

**Figure 5:**
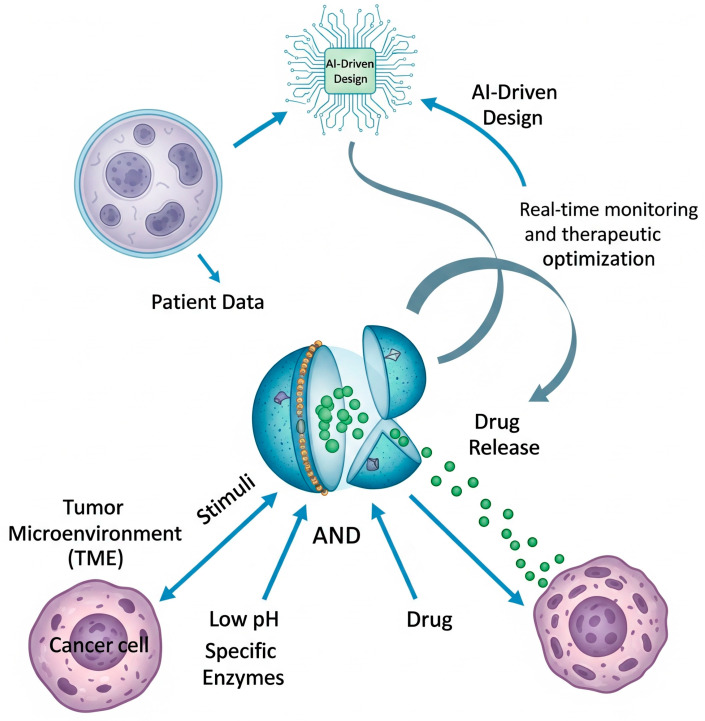
An AI-driven, closed-loop paradigm for personalized nanomedicine. The system uses patient-specific data to design a multi-stimuli responsive nanocarrier, which is then monitored in real-time to optimize treatment.

Despite the translational hurdles, the field of smart drug delivery continues to advance at a rapid pace (Figure 5), pushing towards systems with even greater levels of sophistication and intelligence [99]. To enhance the specificity of drug release beyond what a single trigger can offer, researchers are developing multi-stimuli responsive and logic-gated systems. A significant portion of nanocarrier design focuses on AND logic gates, which require the simultaneous presence of both the acidic TME and a tumor-specific enzyme to trigger drug release. This ‘double-check’ strategy is crucial for reducing off-target effects, ensuring that the payload is only released under tumor-specific conditions while sparing healthy tissues [100]. Furthermore, these systems can be programmed for sequential activation to overcome multiple biological barriers in a specific order. A nanoparticle might first shed its protective PEG layer in response to MMPs to enhance tumor penetration, then use a pH-responsive element to escape the endosome, and finally, leverage a redox-sensitive linker to release its drug within the cytoplasm, executing a series of pre-programmed actions [101].

The design space for these complex biomaterials is astronomically large, making traditional trial-and-error discovery inefficient. Consequently, AI and machine learning (ML) are emerging as transformative tools to accelerate the design–build–test cycle [102]. Emerging AI applications in materials research, such as AI-driven virtual library screening, property prediction, and candidate prioritization pipelines, have greatly accelerated the identification of promising materials while reducing the time and resources required compared to traditional experimental approaches. ML models can also analyze complex formulation data to optimize manufacturing processes, identifying the key parameters that control particle size, drug loading, and release kinetics, thereby reducing the need for costly and time-consuming experiments [103].

The ultimate vision is to leverage these advancing technologies to achieve truly personalized nanomedicine, moving beyond “one-size-fits-all” therapies [104]. This future paradigm may involve analyzing a patient’s tumor biopsy to create a detailed profile of its unique microenvironment—its precise pH, specific enzyme signature, and receptor expression. This data could then be fed into an AI model to design a bespoke smart DDS optimized for that individual’s specific pathological triggers (Figure 5). By incorporating a theranostic component, clinicians could then monitor the custom DDS in real-time, creating a closed-loop therapeutic system that is continuously optimized for maximum efficacy and minimum toxicity for each specific patient [105].

## 6. Conclusions

Biomolecule-based smart DDSs represent a promising advance in medicine, offering the potential to improve upon conventional chemotherapeutic approaches by providing precise, intelligent, and personalized therapeutic interventions. By harnessing the inherent programmability and biocompatibility of nature’s building blocks—nucleic acids, peptides, proteins, and polysaccharides—scientists are creating materials that can sense the subtle biochemical cues of disease and respond by delivering potent therapies with unprecedented spatiotemporal control. The design principles are now well-established, with a sophisticated toolkit of endogenous and exogenous triggers available to actuate release, and a diverse array of biomolecular architectures capable of performing complex tasks, from targeted delivery and overcoming drug resistance in cancer to providing localized, on-demand treatment for inflammatory diseases and enabling a new generation of non-viral gene therapies.

However, the path from the laboratory to the clinic remains a formidable one. The very complexity that makes these systems so powerful also creates significant hurdles in manufacturing, quality control, biocompatibility assessment, and regulatory approval. The translational chasm between the thousands of successful preclinical studies and the small number of clinically approved nanomedicines is a stark reminder of the challenges that lie ahead. Yet, the outlook is one of profound optimism. The field is not only maturing in its understanding of the complex nano-bio interactions that govern in vivo performance but is also on the cusp of being revolutionized by enabling technologies. The development of multi-stimuli responsive and logic-gated systems is pushing the boundaries of what is possible, moving from simple response to true nanoscale intelligence. Most significantly, the integration of AI and ML into the design process promises to provide the tools needed to master the immense complexity of these systems, accelerating the discovery, optimization, and personalization of nanomedicines. The continued, synergistic advancement in material science, molecular biology, and computational power provides a clear and exciting trajectory toward a future where intelligent, biomolecule-driven systems will be a cornerstone of precision medicine, offering safer, more effective, and tailored treatments for humanity’s most challenging diseases.

## Data Availability

Not applicable.
